# Short-Chain Fatty Acids, Maternal Microbiota and Metabolism in Pregnancy

**DOI:** 10.3390/nu13041244

**Published:** 2021-04-09

**Authors:** Maciej Ziętek, Zbigniew Celewicz, Małgorzata Szczuko

**Affiliations:** 1 Department of Perinatology, Obstetrics and Gynecology, Pomeranian Medical University in Szczecin, Broniewskiego 24, 71-460 Szczecin, Poland; sekr.perinat@spsk1.szn.pl; 2 Department of Human Nutrition and Metabolomics, Pomeranian Medical University in Szczecin, Siedlecka 2, 72-010 Police, Poland; malgorzata.szczuko@pum.edu.pl

**Keywords:** lipids, SCFA, pregnancy, gestational diabetes mellitus, gestational hypertension, microbiota, preeklampsia

## Abstract

Short-chain fatty acids (SCFAs), as products of intestinal bacterial metabolism, are particularly relevant in the diagnosis of intestinal dysbiosis. The most common studies of microbiome metabolites include butyric acid, propionic acid and acetic acid, which occur in varying proportions depending on diet, age, coexisting disease and other factors. During pregnancy, metabolic changes related to the protection of energy homeostasis are of fundamental importance for the developing fetus, its future metabolic fate and the mother’s health. SCFAs act as signaling molecules that regulate the body’s energy balance through G-protein receptors. GPR41 receptors affect metabolism through the microflora, while GPR43 receptors are recognized as a molecular link between diet, microflora, gastrointestinal tract, immunity and the inflammatory response. The possible mechanism by which the gut microflora may contribute to fat storage, as well as the occurrence of gestational insulin resistance, is blocking the expression of the fasting-induced adipose factor. SCFAs, in particular propionic acid via GPR, determine the development and metabolic programming of the fetus in pregnant women. The mechanisms regulating lipid metabolism during pregnancy are similar to those found in obese people and those with impaired microbiome and its metabolites. The implications of SCFAs and metabolic disorders during pregnancy are therefore critical to maternal health and neonatal development. In this review paper, we summarize the current knowledge about SCFAs, their potential impact and possible mechanisms of action in relation to maternal metabolism during pregnancy. Therefore, they constitute a contemporary challenge to practical nutritional therapy. Material and methods: The PubMed database were searched for “pregnancy”, “lipids”, “SCFA” in conjunction with “diabetes”, “hypertension”, and “microbiota”, and searches were limited to work published for a period not exceeding 20 years in the past. Out of 2927 publication items, 2778 papers were excluded from the analysis, due to being unrelated to the main topic, conference summaries and/or articles written in a language other than English, while the remaining 126 publications were included in the analysis.

## 1. Introduction

Short-chain fatty acids (SCFAs) are the main end products of the metabolism of bacteria attached to surfaces of the intestinal lumen. SCFAs are carboxylic acids with aliphatic tails of 1–6 carbons [1]. SCFAs include: acetic acid (C2: 0), propionic acid (C3: 0), butyric acid (C4: 0), valeric acid (C5: 0) and caproic acid (C6: 0), of which butyric acid, acetic acid and propionic acid are the most abundant in human organisms [1,2,3]. SCFAs provide about 10% of the caloric requirement in humans [4], affecting inter alia, brown adipose tissue, mitochondrial functions in the liver and appetite control [1]. Most of the SCFA produced in the gut lumen (90–95%) is absorbed through the intestinal mucosa, and 5% is excreted in the feces [3]. Fermentation of carbohydrates (polysaccharides and oligosaccharides) in the proximal part of the colon by saccharolytic bacteria leads to the linear production of short-chain fatty acids (n-SCFAs), H_2_ and CO_2_, while the fermentation of amino acids or proteins is associated with branched SCFAs (BSCFAs), H_2_, CO_2_, CH_4_, phenols and amines production [5]. The role and impact of SCFAs on metabolism in pregnancy are not recognized; therefore, the authors decided to collect the available information, review it and systematize the current state of knowledge.

### 1.1. Division of Short-Chain Fatty Acids (SCFAs)

The amount of SCFAs produced in the gastrointestinal tract varies and depends on the diet, type and amount of the host’s microbiome, as well as the time of residence in the gastrointestinal tract [6]. The synthesis of acids with a longer carbon chain can take place from acetic acid and propionic acid by extending it. On the other hand, BSCFAs, which mainly include isovaleric and isobutyric acids, are produced during the fermentation of branched amino acids (valine, leucine, isoleucine) by the intestinal microflora and are present in much smaller amounts than n-SCFAs [7]. In humans, the species Bacteroides and Clostridium are mainly responsible for the fermentation process of branched chain amino acids [8]. Differences in the distribution of BSCFAs concentrations in the human large intestine were observed, with a tendency to higher concentrations in the proximal part of the colon compared to the distal part and feces [9]. Both the metabolism of BSCFAs and the types of intestinal microbial populations involved in their production, as well as their role in the human body, are not yet sufficiently understood.

### 1.2. The Diet and SCFAs Synthesis

In studies conducted on intestinal tissues in vitro, a positive relationship was observed between a high-protein diet and a low-carbohydrate diet, with a higher level of BSCFAs concentrations in the colon of piglets [10]. In a randomized crossover study by Hald et al. [11], it was reported that the concentrations of isobutyrate (*p* = 0.05) and isovalerate (*p* = 0.03) in humans with metabolic syndrome decreased in response to the arabinoxylan and resistant starch enriched diet. These results indicate a reduced protein fermentation [11]. Lower BSCFAs levels were also observed when comparing high-carbohydrate/low-protein (including high fiber) diets with high-protein/low-carbohydrate diets in humans with metabolic syndrome [9]. In the human adult population, a negative correlation was shown between the consumption of insoluble dietary fiber and the level of BSCFAs in the feces [9]. Hashemi et al. [12] demonstrated in a study conducted on rats that pea fiber supplementation improves glycemia and alters the composition of the gut microbiota and the profile of short-chain fatty acids in blood serum as well. The investigators concluded that a high fat diet (HFD) supplemented with raw and cooked pea seed coat fractions may have a protective role against HFD-induced alterations in gut microbiota [12]. David et al. [13] reported that the animal-based diet is related both to an increased abundance of bile-tolerant microorganisms such as Alistipes, Bilophila and Bacteroides, and to decreased levels of Firmicutes that metabolize dietary plant polysaccharides (Roseburia, Eubacterium rectale and Ruminococcus bromii). Consumption of plant-based protein was reported to increase Bifidobacterium, Lactobacillus and Enterococcus, and to decrease Escherichia coli. In addition, the changes in gut microbiome composition followed by a protein diet were observed to produce SCFAs. Supplementation of a fiber-rich diet is known to increase the Bifidobacterium, Prevotellaceae and Lachnospiraceae, while decreasing the abundance of Porphyromonadaceae and Lactobacilli [13].

The supply of substrates in the diet is the most important variable for their synthesis [5], and the gut microbiome can rapidly respond to altered diet [13]. It is estimated that 5% to 20% of dietary starch is not absorbed by the human gut [5]. Studies in mice have shown that the mother’s diet before and during pregnancy had a significant effect on the intestinal microflora [14]. Probably, the intestinal maternal changes contribute to both the unfavorable metabolic adaptation of the mother and the placenta, which, by changing the glucose metabolism in the fetal liver, may increase the risk of metabolic disorders of the newborn. SCFAs produced by anaerobic bacteria also play a key role in regulating gut immunity. Studies by Vetrani et al. [15] showed that the whole grain diet (mainly wheat) used for 12 weeks in humans is related to an increase in the concentration of propionic acid in the fasting plasma. In conditions of dietary fiber restriction in the large intestine, its luminal pH increases, causing the disappearance of butyrate-producing bacteria in favor of the dominant acetic acid and propionic acid-producing Bacteroides [16]. In physiology, an increase in SCFAs’ concentrations is noticeable when following a diet with high-fiber soluble products, while the production of SCFAs is reduced with use of a fiber-restricted or high-fat diet [17,18,19]. Significant changes in the intestinal microflora were observed in humans depending on the diet used [20]. A comparative analysis in humans with normal body weight and those with obesity, fed the same diet for three days, showed an increase in energy retention by ~630 kJ (~150 kcal) in obese people, as well as an increase in the number of Firmicutes strains and a decrease in the number of Bacteroidetes fecal SCFAs; concentration in response to various diets [20]. SCFAs’ metabolites can also affect the level of the satiety hormone, which can lead to disturbances in eating. In studies by Aronsson et al. [21], mice fed a high-fat diet supplemented with Lactobacillus paracasei showed a significant reduction in adipose tissue, accompanied by an increased level of circulating fasting-induced adipose factor (FIAF). On the other hand, increased levels of Bacteroides and decreased Firmicutes, obtained by a diet enriched with soluble fiber, are associated with an increase in SCFAs’ levels [17].

### 1.3. Occurrence and Proportion of SCFAs

The molar ratio of SCFAs in the human colon shows relatively constant proportions and is 60:20:20 for acetic acid, propionic acid and butyric acid, respectively [1,22,23,24]. The percentage of particular SCFAs may change due to factors disturbing the intestinal microflora [3,25]. The concentration of SCFAs varies depending on their gastrointestinal locations: in the distal part of the colon, it is lower (20–70 mM), and it is higher in the proximal part (70–140 mM), which is related to the increasing availability of carbohydrates and water in the proximal part of the colon compared to the distal one [5]. Different intestinal bacteria secrete different amounts of SCFAs: the gram-negative bacteria (Bacteroides) mainly produce acetic acid and propionic acid, while gram-positive bacteria (Firmicutes) mainly produce butyric acid [6]. SCFAs as products of bacterial anaerobic fermentation of dietary fiber in the large intestine act as signaling molecules via specific G-protein coupled receptors (GPCRs), transferring information between the microbiota and the immune system [3]. GPCRs (also known as free fatty acid receptors—FFARs) include seven transmembrane domains and have the ability to bind ligands in the extracellular environment (e.g., fragrances, hormones, neurotransmitters, chemokines, sugars, lipids, proteins) [1]. The variants of the FFARs, each encoded by a separate gene, and including FFAR1 (GPR40), FFAR3 (GPR41), FFAR2 (GPR43), GPR84 and FFAR4 (GPR120), were shown to be activated by free fatty acids (FFA). Of these, mainly FFAR3 and FFAR2 are activated by SCFAs [1,26,27,28]. Both the SCFAs and the receptors through which they act show different degrees of mutual affinity. For FFAR2 receptors, the highest affinity is followed by SCFAs C2 and C3, then C4 and C5 and C1. On the other hand, SCFAs C3, C4 and C5 show high affinity for FFAR3 receptors, followed by C2 and C1; binding strength is listed here in decreasing order [29,30]. Although the regulatory activity of the GPR43 receptor is not fully understood, its role in maintaining energy homeostasis is documented [28]. The presence of GPR43 is demonstrated in adipose tissue [31,32], intestines [33,34] (regulatory T cells—Treg cells), other immune tissues [31,32] as well as in alpha and beta islet cells [35,36] and monocytes, neutrophils and eosinophils [32,37]. SCFAs induce colon Treg cells, thereby promoting the anti-inflammatory response pathway [37,38,39] and controlling inflammation by limiting the proliferation of CD4+ effector T cells. Treg cells are a specific subset of T cells with a potential role in inhibiting and maintaining inflammatory homeostasis [40]. SCFAs are a natural histone deacetylase (HDAC) protease inhibitor, and their inhibitory effect depends on their concentration [1] and the type of SCFA. HDAC is a class of enzymes that removes acetyl groups from ε-N-acetyl lysine on histones, enabling tighter winding of DNA strands around histone proteins [41], thereby modifying the structure of the chromosome and regulating gene expression [1]. The effect of GPR43 stimulation by SCFAs is the inhibition of cAMP secretion [1], activation of extracellular signal-regulated kinase, increase of intracellular Ca^2+^ levels and the activation of mitogen-activated protein kinase (MAPK) [28,31,32]. Intake of dietary fibers in a high-fiber diet leads to SCFAs’ production in the gut, acting as GPR43 ligands. Activation of GPR43 via SCFAs induces suppression of insulin signaling in adipocytes, which consequently leads to inhibition of fat accumulation in adipose tissue with the preferential use of unincorporated lipids and glucose in muscle tissue [1]. SCFAs, especially butyric acid [42], can inhibit the expression of cytokines Il-6, Il-1β and TNFα, thus demonstrating anti-inflammatory effects [43]. GPR43, acting as a sensor of excessive energy supply in the diet, controls the energy use of the body, modulates its metabolic homeostasis and, consequently, may have a potential therapeutic role in the treatment of metabolic disorders such as obesity and type 2 diabetes [28]. The metabolic effects of SCFAs are also mediated through the nervous system by increasing the levels of leptin and insulin, hormones that stabilize feelings of satiety, secreted by adipocytes and the pancreas [27]. An interesting observation from our research conducted on pregnant women is that linear SCFAs are involved in the regulation of carbohydrate metabolism independently of obesity. Dietary fiber-derived SCFAs are involved in the regulation of carbohydrate metabolism, while BSCFA, isobutyric, isovaleric and isocaproic acids from fermentation of undigested protein reaching the colon do not have such a metabolic effect [44].

## 2. Characteristics of SCFAs

### 2.1. Acetic Acid

Acetic acid is the most abundant SCFA in the human colon [5] and accounts for over 50–60% of SCFAs content [45,46]. It is produced by intestinal bacteria of the genus Bifidobacteria and Lactobailli, but also *Akkermansia muciniphila* [47,48] and *Pravotella* spp. *Ruminococcus* spp. [49]. Acetic acid production is mediated by homoacetogenic bacteria or gastrointestinal acetogens capable of producing acetate from H_2_ and CO_2_. Its sources in the diet are dairy products, pasta, bread, eggs, salt substitutes, coffee and its substitutes, processed meat and smoked fish, as well as ethanol and vinegar [22]. After a meal, the acetogenic fibers ferment and thus increase acetic acid production in the proximal colon. The involvement of the increased abundance of the Bifidobacteria species effects in acetates further changes, and affects butyric acid production, which is an important source of energy. Acetic acid acts by binding to GPCRs (FFAR2, FFAR3) present in the human colon [34,50], but it is also expressed at the mRNA level in various insulin-sensitive tissues such as adipose tissue [26], skeletal muscle, liver [51] and pancreatic beta cells [52]. Acetic acid is the main substrate in the synthesis of cholesterol [5]. It can be converted into acetyl-CoA and included in the tricarboxylic acid cycle in peripheral tissues [22]. The presence of Acetyl-CoA synthetase in the cytosol of adipose tissue adipocytes determines the use of acetic acid in the lipogenesis process [5]. Acetic acid increases the synthesis of fatty acids via epigenetic mechanisms (histone acetylation) [53]. Its metabolic activity is also due to increasing the oxidative capacity (e.g., liver and skeletal muscles) by influencing the phosphorylation of 5′ activated protein kinase AMP (AMPK) [54]. Acetic acid is also an important factor in pH regulation, thus maintaining an acidic environment in the intestinal lumen [22]. Schwiertz et al. [46] evaluated differences within the human intestinal microbiota and fecal SCFA concentration of lean and obese persons. It was shown that the mean total concentration of SCFAs in the stool of obese subjects is over 20% higher when compared to lean subjects, and the difference is statistically significant [46]. The largest increase was found for propionic (41%), followed by butyric (28%), valeric (21%) and acetic acid (18%) [46]. There was also a positive correlation between acetic acid and anthropometric (BMI) and biochemical parameters (fasting glucose and 60 min in the OGTT test, as well as insulin and HbA1c levels) in the group of overweight or obese (before pregnancy) pregnant women [44].

### 2.2. Propionic Acid

The main producer of propionic acid is bacteria of the genus Bacteroides, Fimicutes and Lachnospiraceae. The latter can also produce butyric acid, depending on the substrate. Although this SCFA is less understood, its activity is known to be associated with beneficial health effects. The clinical effect of propionic acid on the lipid metabolism manifests itself in the form of a reduction in cholesterol concentrations and a reduction in fat storage; it also has anti-cancer and anti-inflammatory activity. The participation of propionic acid in the process of cholesterol synthesis in the liver tissue was also previously demonstrated [5]. In a double-blind randomized trial, 20 women were given either 7.5 g of sodium propionic acid daily for a period of seven weeks or were supplemented with dibasic calcium phosphate (placebo). Although propionic acid supplementation did not lower total serum cholesterol, an increase in high-density lipoprotein cholesterol (HDL-C) concentrations and an improvement in glucose tolerance and insulin sensitivity were observed [5]. The inhibitory effect of propionic acid on gluconeogenesis may result from the activity of metabolic intermediaries, methymalonyl-CoA and succinyl-CoA, which are specific inhibitors of pyruvate carboxylase [5]. Propionic acid is synthesized by two pathways: the dicarboxylic acid pathway and the acrylate one. In the first way, CO_2_ is bound to succinate, which is then decarboxylated, while in the second, lactate and acrylate are involved [5]. The increase in the level of propionic acid was positively associated with the plasma glucose concentration and HbA1 in the group of obese pregnant women, and with an increase in body weight and BMI in normal-weight women, both before and during pregnancy [44].

### 2.3. Butyric Acid

Butyric acid is produced by intestinal bacteria of the genus *Faecalibacterium prausnitzii*, *Eubacterium rectale* and *Roseburia* spp. [45]. Butyric acid, although produced in the smallest amount as compared to acetic acid and propionic acid, has a significant beneficial effect on cellular energy metabolism and intestinal homeostasis, being the main source of energy for colonocytes [55,56]. The substrate for the production of butyric acid is dietary fiber, which undergoes two metabolic pathways as a result of bacterial fermentation. First, there is phosphorylation of butyryl-Coenzyme A (butyryl-CoA) to butyrylphosphate and then transformation to butyric acid by kinase [57]. In the next step, the CoA moiety of butyrylCoA is under the influence of the butyryl-CoA enzyme: Acetate CoAtransferase is converted via acetic acid to butyric acid and acetyl CoA [58]. The stimulating effect of butyric acid on the production of leptin in adipocytes and the induction of GLP-1 by L-cells of the intestine were demonstrated. It also influences the thermogenesis process and the increase in fatty acid oxidation [59]. An important role in modulating the immune and inflammatory response by influencing the release of cytokines and chemokines was also found [59]. Chang et al. [60] demonstrated that butyric acid, by the inhibition of HDAC, inhibits the production of pro-inflammatory cytokines (IL-6 and IL-12) induced by endotoxin-induced Gram-negative bacteria—lipopolysaccharide (LPS), by way of GPCRs’ independent pathway. It was demonstrated that in HeLa cell lines in colorectal cancer, butyric acid exhibits a stronger HDAC inhibitory activity compared to propionate [1]. The inhibitory effect of butyric acid on HDAC protease promotes the intensification of the H3 reaction of histone acetylation at the Foxp3 gene locus and induces Treg in the intestine [38,39,45]. Among SCFAs, butyric acid most strongly inhibits HDAC activity [45], which thus promotes the inhibition of cell proliferation, induction of their differentiation or apoptosis. Finally, butyric acid has anti-carcinogenic properties. The anti-inflammatory effect of butyric acid is mediated by inhibiting the activation of a transcription factor, known as nuclear factor kappa-light-chain-enhancer of activated B cells (NF-κB) [61]. NF-κB regulates the expression of many genes involved in the inflammatory process as well as immunity [61]. Butyric acid plays a key role in the downregulation of pro-inflammatory effectors of lamina propria macrophages and determines the expression of cytokines in T cells [60,62]. The absorption of SCFAs through the apical membrane of colonocytes occurs passively by diffusion in the case of the non-dissociated form or by active transport in the case of the dissociated form. Active transport occurs with the participation of two SCFAs’ transporters: the human colonic monocarboxylate transporter (MCT) isoform 1 (MCT1), coupled with the transmembrane H^+^ gradient [63], and the human solute carrier transporters, also known as the sodium-coupled monocarboxylate transporter (SMCT) [45]. In the case of butyric acid, the transport is mediated mainly through MCT in an acidic environment, with an optimal pH of 5.5 [60]. It is possible that butyric acid contributes to obesity by increasing the synthesis of lipids from acetyl-CoA or ketone bodies via the β-hydroxy-β-methylglutaryl-CoA pathway [64].

### 2.4. Uncommon SCFAs

As the diversity of the gut microbiota increases, other SCFAs are produced, such as valeric and caproic acid, and branched SCFAs: isobutanoic, isovaleric and isocaproic acid, which is considered a marker of colonization with Clostridium difcile strains [65]. There are few reports on the role and metabolic pathways of long-chain and branched SCFAs during pregnancy. The concentration of propionic and linear caproic acids may be an important critical point in maintaining lower anthropometric parameters during pregnancy, because in the group of obese pregnant women, statistically significantly higher concentrations of propionic acid and a reduced content of caproic acid were demonstrated compared to the control group [44]. In the above study, a tendency of reduction in the concentration of linear valeric acid in the group of obese pregnant women was also observed. Stool concentrations of some uncommon SCFAs in children aged 1 (*n* = 139) and 4 (*n* = 53) varied and were shown to be associated with food allergy occurrence. The observed decrease in fecal acetic and propionic acids concentrations in the period from 14 years of age was accompanied by an increase in valeric acid concentrations, while the concentrations of iso-butyric, valeric and iso-valeric acids at four years of age showed an inverse relationship with the development of food allergies [66]. It was demonstrated that valeric acid, together with propionic and butyric acid, inhibits the inflammatory process by inhibiting both NF-κB transactivation and interleukin-8 secretion [67].

## 3. Changes in the Intestinal Microbiota during Pregnancy

Dietary fiber provided with the diet plays a key role in shaping the human microbiome. The lack of presence of adequate enzymes acting on the carbohydrates in the upper gastrointestinal tract determines the fermentation processes in the lower intestine [68]. The adult human gut is colonized by at least 1800 genera and approximately 15–36,000 species of bacteria. The majority of all isolated microorganisms (94–98%) belong to four groups of bacteria: Firmicutes (64%), Bacteroidetes (23%), Proteobacteria (8%) and Actinobacteria (3%). The remaining (about 2%), although not numerous, constitute a group of very diverse taxonomies [69]. Pregnancy has been shown to influence the composition of the maternal gut microbiome, which differs in the first and third trimester of gestation [70]. Undigested in the upper gastrointestinal tract, carbohydrates from dietary fiber pass intact to the large intestine, where they are fermented by the anaerobic microbiota [45]. A plant-based diet has a particularly beneficial effect on the development of microbiota, where the low content of saturated fatty acids and high polyunsaturated fatty acids concentrations have a positive effect on the lipid profile [71]. During the first trimester of pregnancy, the composition of the gut microbes is similar to that of healthy, non-pregnant women. However, from the first to the third trimester, the composition of the intestinal microflora changes significantly [72]. The intestinal microflora in women in the third trimester of pregnancy show higher proportions of phylum Proteobacteria strains associated with inflammation [70]. Between the first to the third trimester, the number of Bifidobacteria, Proteobacteria and lactic acid-producing bacteria increases, while the number of butyrate-producing bacteria is decreased [70]. In studies in mice implanted with intestinal microflora from women in the third trimester of gestation, increases in body weight and insulin resistance were observed, reflecting the diabetogenic changes observed in pregnant women. The above results may indicate that changes in the intestinal microflora during pregnancy contribute to the occurrence of metabolic changes characteristic of pregnancy, with an increase in inflammatory markers and energy content [70]. One of the proposed mechanisms by which the intestinal microflora influences the weight gain of a pregnant host woman is increased glucose and fatty acid absorption, increased secretion of adipocytic factor in the fasting state, induction of catabolic pathways and stimulation of the immune system [70]. The essential changes in the gut microbiota during pregnancy include an increase in the number of bacteria and profound modifications to the composition of the gut microbiota, mainly in late pregnancy [70]. The evolution of changes in the microbiome in pregnancy includes an increased abundance of Actinobacteria and Proteobacteria phyla strains, as well as a reduction in individual bacterial diversity [70]. The persistence of microbiota-induced changes in the composition of various factors (dysbiosis) may be associated with detrimental long-term effects, leading to diseases such as obesity, enteritis, diabetes and metabolic syndrome in the host organism and offspring [73]. It is possible that human intestinal microflora play a role in the regulation of nutrient metabolism, and dysbiosis may be associated with an increased acquisition of energy resources [74].

## 4. Change in SCFAs Content during Pregnancy

**Figure 1:**
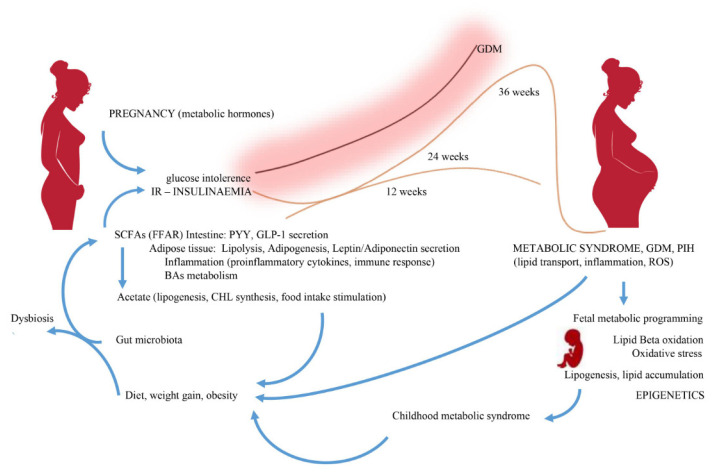
The influence of changing the microbiome and their metabolites on the metabolism of a pregnant woman. IR—insulin resistance; SCFAs—short-chain fatty acids; FFAR—free fatty acid recep-tor; Bas—bile acids; GDM—gestational diabetes mellitus; PIH—pregnancy induced hy-pertension; GLP-1—glucagon-like peptide-1; PYY—peptide YY; ROS—reactive oxygen species.

During pregnancy, the gut microbiome changes in women with diet-induced obesity. Intestinal dysbiosis is accompanied by inflammatory processes and, consequently, altered levels of SCFAs and the expression of receptors through which they exert their metabolic actions. Maternal microbial populations show statistical differences between obese and lean individuals [75]. In obese individuals, a reduction in the number of bacteria of the genera Clostridiales, Ruminococcaceae and Lachnospiraceae, which mainly produce butyrate, is most evident. Assessment of SCFAs’ concentration in the cecum has shown that pregnancy increases the abundance of acetic and propionic acid, and also tends to increase the levels of butyric and caproic acid [76]. Acetic acid has been found to be the dominant SCFA in both pregnant women and their infants [77]. The highest concentrations of acetic acid were observed in infants aged three months, while the lowest were observed after 12 months [78]. Increased maternal serum SCFAs’ levels may positively affect maternal weight gain, glucose metabolism and levels of various metabolic hormones [78]. Serum acetic acid levels were also shown to be associated with maternal weight gain and maternal adiponectin levels, while serum propionate levels negatively correlated with maternal leptin levels [78]. Adipokines (adiponectin and leptin), as adipocyte-specific hormones, play an important role in the metabolic response during pregnancy, contributing to the regulation of satiety, insulin resistance and, consequently, obesity [79]. SCFAs are active at the cellular level by increasing free fatty acids’ oxidation, mitochondrial activity in muscle and brown adipose tissue [80]. In the physiological conditions, one of the mechanisms by which the body is protected against lipid overload and diet-induced obesity is elevated FIAF levels [81]. FIAF inhibits the action of lipoprotein lipase (LPL), the enzyme responsible for energy storage in the form of fat [82]. Blocking the expression of this factor by abnormal gut microflora may therefore promote fat storage through increased LPL activity. Acetic acid supplementation in a mouse model showed an effect on the immune system of fetuses, with increased production of CD4+ T cells after birth, increased expression of protein forkhead box P3 (Foxp3) and protection of specialized thymic epithelial cells with expression of the autoimmune regulator (AIRE) gene, which is necessary for autoimmune regulation and the formation of Treg cells in early life [83]. Among the SCFAs that influence the epigenome, butyric acid in particular is recognized as a potent inhibitor of histone deacetylase [84]. Butyric acid exhibits anti-inflammatory activity by promoting Treg cell differentiation and increasing histone H3 acetylation, which corresponds to increased Foxp3 mRNA expression. Expression of Foxp3 is a key element in the regulatory function of CD25 (+) T cells in mice [85]. In pregnant mice, supplementation with butyric acid was associated with a decrease in maternal pro-inflammatory factors TNFα and IL-1β, as well as weight gain and an increase in blood glucose, insulin, triacylglycerol and cholesterol levels [86]. Dietary butyric acid supplementation alleviated obesity caused by a high-fat diet and decreased pancreatic beta cell dysfunction, which was manifested by increased insulin storage, beta cell size, their mass and accelerated apoptosis [86]. Obesity in pregnancy is not only accompanied by decreased production of butyrate, but also of Beta defensin 3, which significantly changes the properties of the intestinal barrier. The effect of butyrate on the intestinal barrier is through the transcriptional regulation of tight junctions protein claudin-1, the induction of occludin and the redistribution of zonulin-1 in cell membranes [87]. It was shown in mice that propionate entering the fetal circulation in the embryo mediates not only the control of insulin levels through GPR43 signaling, but also influences the development of the sympathetic nervous system through GPR41 signaling [88]. In mice, SCFAs, in particular propionate, influence the embryo development via receptors GPR41 (development of the nervous tissue and the heart’s conduction system) and GPR43 (development of the enteroendocrine system and pancreatic beta cells). Propionic acid can be a source of energy through its use in the synthesis of lipids and glucose. The presence of uteroplacental GPR41 and GPR43 receptors and their role in inflammatory processes during pregnancy and delivery may be the pathway by which SCFAs influence fetal metabolic programming [89,90]. It was shown in an animal model (mice) that exogenously supplemented SCFAs reduce hepatic fat reserves and inhibit the expression of genes related to lipid synthesis [91]. The energy resources of a pregnant woman are modulated, inter alia, by exogenous food consumption and de novo synthesis of lipids and glucose from SCFAs (Figure 1). In an animal model, SCFAs from the maternal gut microbiota were sensed via GPR41 and GPR43 receptors in the fetus in sympathetic nervous tissue, gastrointestinal tract and pancreas [88]. Obesity in pregnancy promotes pro-inflammatory TLR signaling and induces pro-inflammatory macrophages in placental tissue. As a consequence, there is placental hypoxia and a pro-angiogenic reaction with an increase in the level of immunoreactive proteins such as VEGF (vascular endothelial growth factor) and CD31 (cell adhesion molecule-1). The above changes in placental function in obese pregnant individuals affect the fetal functions and further newborns’ metabolic programming, who are at higher risk of IR, diabetes mellitus and fatty liver disease [14]. The inflammatory processes observed in the placenta of pregnant obese animals—hypoxia increased vascular density, reduced placenta maturity—significantly impair its nutritional function, which determines the impaired metabolic development of the fetus. An additional factor impairing the fetal energy metabolism are the changes in the fetal liver function observed in maternal obesity. In an animal model (female mice), decreased hepatic transcripts of the enzymes pyruvate carboxylase and phosphoenolpyruvate carboxykinase [75] were found. Thus, fetal metabolic programming results from changes in the levels of key liver enzymes and transcription factors that regulate fetal gluconeogenesis.

Changes in diet, obesity and excessive weight gain are associated with changes in the gut microbiome. Therefore, bacteria-released lipopolysaccharides and subsequent endotoxemia lead to dysbiosis. The inflammatory process is activated with the release of pro-inflammatory cytokines. Changes in the SCFAs’ synthesis proportions occur. Acetate, absorbed by liver cells, leads to lipogenesis, while stimulating the food intake. In contrast, propionate and butyrate show protective features against diet-induced obesity. Under normal conditions, SCFAs inhibit insulin signaling in adipocytes, reducing fat storage. Energy homeostasis is regulated by FFAR receptors. FFAR-mediated stimulation of GLP-1 and PYY secretion by SCFAs leads to a reduction in insulin secretion. Lipids and carbohydrates stimulate the secretion of PYY, the main factor suppressing appetite after a meal, and GLP-1, which stimulates insulin secretion to prevent peri prandial increases in glycaemia. A maternal high-fat diet, obesity, abnormal SCFAs proportions, hyperglycemia, hyperinsulinemia, IR and hyperlipidemia lead to increased transplacental lipid and glucose transport, triggering inflammatory stress and beta-oxidation in the fetus. As a result, fetal lipogenesis and fat accumulation induce symptoms of metabolic syndrome in neonate.

## 5. Association of SCFAs with Hypertensive Disorders and Diabetes Complicating Pregnancy

### 5.1. The Role of SCFAs in Arterial Hypertension

Hypertension (HA) is shown to be clearly related to obesity. Obesity is affected by the composition of the gut microbiome alteration, which may also contribute to hypertension and its complications, potentially by an increase in the concentration of low-grade inflammation response, altering endothelial function, which can impact the host’s blood pressure [92]. Another mechanism by which a disturbed gut microflora can influence blood pressure is the synthesis of neurotransmitters acting in the autonomic nervous system, by bacteria belonging to the Streptococcus, Escherichia, Lactobacillus and Bifidobacterium strains [93]. Shifting the proportions of these bacteria, which alter the production of SCFAs, may affect vascular tone and contribute to the development of HA, as demonstrated by studies in an animal model (Dahl rats) [94]. A negative correlation between Bacteroides and blood pressure, body weight and fat mass was previously reported [90]. The host’s inflammatory reaction in response to changes in the gut micro-biota alters endothelial function, which can affect the host’s blood pressure. In studies on rats with HA, the gut microbiota had a decreased Bifidobacteria abundance and fewer SCFA-producing bacteria [92]. Moreover, the authors of this study observed quantitative (less quantity) and qualitative (less diversity) changes in the microbiome in HA individuals with a higher ratio of Firmicutes to Bacteroidetes. SCFAs produced by bacteria may not only have a direct influence by vasodilating blood pressure, but also an indirect influence through the plasminogen activator inhibitor -1 (PAI-1). Under laboratory conditions, it was shown that n-Butyrate leads to an increase in PAI-1 mRNA in human hepatocyte cultures [95]. The impaired function of the vascular endothelium in HA induces an inflammatory response manifested by an increase in C reactive protein (CRP), which then induces PAI-1. In overweight and obese women, PAI-1 activity is found to be inversely proportional to serum adiponectin levels, regardless of visceral adipose tissue [96]. Significant correlations between PAI-1 levels and lipoprotein subfractions indicate a relationship between PAI-1 and lipid metabolism in obesity [96]. There are strong, significant negative correlations between plasma PAI-1 concentrations and low-density lipoprotein, as well as between PAI-1 concentrations and high-density lipoprotein levels [97].

### 5.2. The Associations of SCFAs with Hypertension in Pregnancy

Similar observations were also reported in the group of pregnant women in whom increased arterial pressure, both systolic and diastolic, was associated with altered intestinal microflora composition and butyric acid production since the early course of pregnancy [95]. High blood pressure with signs of damage to another organ system (most often the liver and kidneys) beginning after the 20th week of gestation characterizes a pregnancy complication—preeclampsia (PE). PE is a disorder of widespread vascular endothelial malfunction and vasospasm, remaining a leading cause of maternal and neonatal mortality and morbidity. Although PE occurs in 4–5% of all pregnancies, its pathophysiology is not yet fully understood. Hu et al. [98] found evidence of an association between low serum acetate and subsequent PE in a pregnancy cohort. The authors also demonstrated that the fetal immune consequences of PE are persistent, with visible reduction of thymus Treg frequency beyond infancy and into early childhood, suggesting a long-term implication of PE for immune function in offspring. It is possible that maternal gut microbiota may influence the pathogenesis of PE, while probiotic use may be associated with its reduced risk. A Mediterranean diet and a diet high in fiber promote SCFAs’ production, resulting in a decrease in the incidence of PE [98]. Chang et al. [99] found in patients with PE a significantly decreased GM diversity and altered GM abundance. In the PE group, they observed a decreased abundance of Firmicutes and increased abundance of Proteobacteria. Patients with PE had a lower abundance of *Blautia, Eubacterium_rectale, Eubacterium_hallii, Streptococcus, Bifidobacterium, Collinsella, Alistipes* and *Subdoligranulum*, and a higher abundance of *Enterobacter* and *Escherichia-Shigella*. The authors also concluded that butyrate significantly reduces the blood pressure in hypertensive pregnant women. This observation of direct blood pressure regulation by butyrate suggests its potential role as a therapeutic agent for PE. The increased blood levels of circulating PAI-1 as well as its increased placental expression were observed in pregnant women complicated by HA, including PE and HELLP syndrome (haemolysis, elevated liver enzymes, thrombocytopenia) [92,100]. It is suggested that dietary SCFAs induce the endothelial peroxisome proliferator-activated receptor-γ (PPARγ)-dependent pathway of lipid metabolism. PPARγ plays an essential role in endothelial dysfunction prevention. PPARγ controls endothelial function and blood pressure homeostasis, and by contributing to maternal vascular adaptation, it provides information on how obesity and gestational diabetes may be related to pregnancy-induced hypertension and PE [101]. The interference with endothelial PPARγ may influence vascular dysfunction, inflammation and senescence via mechanisms involving oxidative stress, occurring in PE. Although there are many indications that the intestinal microflora and SCFAs are associated with hypertension, their impact on the course of hypertension in pregnancy and the prediction of PE remain unknown.

### 5.3. The Role of SCFAs in Diabetes Mellitus

SCFAs play an important role in additional energy acquisition coming from undigested foods and in glucose homeostasis. Depending on the diet and the type of SCFA produced, different metabolic pathways are triggered through different receptors. SCFAs modulate host lipids and glucose metabolism through the GPR41 and GPR43 receptor junctions [102]. Although these receptors show over 40% structural similarity, they differ in their ligand binding specificity [103]. SCFAs also increase lipid storage capacity by inhibiting lipolysis mediated by activated GPR43 and thus reducing the concentration of free fatty acids in the blood serum [104]. SCFAs affect the lipid metabolism also by stimulating FIAF and facilitating adipogenesis. In response to the activation of GPR43, the Gi/o protein is stimulated, which activates intracellular signaling pathways, inter alia, inhibiting the production of adenylate cyclase [1]. SCFAs through GPR43 receptors mediate inhibition of the lipolysis process in primary human fat cells [32]. Activation of GPR43 by SCFAs, both in vitro and in vivo, increases the secretion of leptin, which facilitates the absorption of glucose in brown adipose tissue and muscles, and improves hepatic metabolism via Gamma aminobutyric acid and proopiomelanocortin in the hypothalamus [1]. Under the influence of SCFAs, increased glycogen storage and inhibition of the glycolysis process are also observed in these organs [105]. Propionate is the main substrate in the process of gluconeogenesis, while its inhibitory effect on fatty acid synthetase reduces lipogenesis, opposite to acetic acid and butyric acid, which enhance this process. At the endocrine level, SCFAs stimulate the secretion of glucagon-like peptide-1, which enhances insulin secretion and inhibits the release of glucagon in the pancreas [106]. In the studies of Qin et al. [107], in the conditions of limited butyric acid synthesis, an increase in the transmembrane transport of sugars, branched-chain amino acids and the concentration of oxidative stress markers was shown, which suggests that depending on altered microbiota and dietary fiber fermentation, the produced SCFAs have an immunomodulatory effect and affect host energy metabolism. The authors have shown that patients (345 Chinese individuals) with type 2 diabetes had a moderate degree of intestinal dysbiosis, decreased numbers of some butyrate-producing bacteria and a concomitant increase in opportunistic pathogens, as well as decreased resistance to oxidative stress. The reduced amount of SCFAs from dysbiotic gut microbes in humans with diabetes can destroy the intestinal mucosa barrier, thereby increasing microbial translocation, ultimately promoting inflammatory responses [108].

### 5.4. The Associations of SCFAs with Gestational Diabetes Mellitus

The insulin resistance (IR) observed in pregnancy is the result of several factors such as obesity, physical inactivity, placental hormones and genetic and epigenetic changes. Under physiological conditions, IR facilitates the energy resources redirection from the mother to the developing fetus, while pathological maternal IR is associated with neonatal obesity [109]. Since the second half of pregnancy, the IR in organs (liver, adipose tissue and muscles) increases significantly and may reach a severe state, resulting in gestational diabetes mellitus (GDM) [110,111]. One of the mechanisms that may explain pathological IR and the development of GDM in pregnancy is an unhealthy diet that changes the quantitative and qualitative resources of the gut microbiota, resulting in the formation of SCFAs that modulate energy metabolism and induce low-level inflammation [104] (Figure 1). GDM as an isolated disease as well as in combination with maternal overweight/obesity contributes to changes in the composition of intestinal microorganisms, their diversity and disturbed SCFA proportions [90]. These disorders are also seen in neonates and result in increased cytokine levels and changes in the early cytokine response of innate immune cells early in life [75,112]. Considerable accumulation of macrophages in the placenta of obese pregnant women results in the production of pro-inflammatory cytokines and adipokines including interleukin-6, leptin, TNF-α, monocyte chemotactic protein 1 and TLR4 [113]. The inflammatory process in the placental tissue promotes the increased release of free fatty acids into the fetal circulation, disrupting its growth and development [113]. Microbiota disorders in gestational diabetes, especially in the second trimester, are so distinct and characteristic that they can be a predictor of GDM [114]. As the expression of FFAR2 gene is higher in the pancreatic inlets during pregnancy, the effect of SCFAs is more visible. In studies of the importance of GPCR signaling in pregnant mice, female FFAR2-deficient mice developed fasting hyperglycemia and impaired glucose tolerance through impaired insulin secretion compared to non-FFAR2-deficient mice. Increasingly, these relationships have not been observed in non-pregnant individuals [115]. A high-fat diet in women with GDM disrupts the intestinal microbiota, leading to the growth of butyrate-producing bacteria (mainly strains Firmicutes and Faecalibacterium), the excess of which leads to an increase in SCFAs production and exceeding the normal lipid capacity in adipose tissue, resulting in a positive energy balance [116]. SCFAs excess activates the glycolysis and gluconeogenesis pathways with inhibition of the insulin response in peripheral tissues, resulting in diabetes mellitus. Insufficient lipid storage capacity in adipose tissue with a decrease in fatty acid oxidation and increased lipolysis leads to an increase in the concentration of FFA in the circulation, resulting in the storage of lipids in the liver and muscles. It was also shown that with the increase in the number of intestinal strains of the genus Collisella, the level of fasting insulin, C-peptide and triglycerides increases in obese or overweight women at 16 weeks of gestation [117]. The above data show that the type of diet, gut microbiome and regulation of carbohydrate and lipid metabolism may be linked by influencing the levels of metabolic hormones. In particular, serum acetate and propionate seem to be associated with multiple interrelated variables: gestational weight gain, maternal adiponectin levels, maternal leptin levels, neonatal length, and body weight [78]. The total amount of SCFAs is higher in obese compared to lean [46], while obese treated individuals exhibit reduced fecal SCFAs [118], suggesting that SCFAs regulate host carbohydrate and lipid metabolism and may contribute to the obesity phenotype. SCFAs can have an effect by enhancing glycolysis/gluconeogenesis pathways and inhibiting insulin signaling in peripheral tissues, resulting in hyperglycemia in pregnancy and diabetes. Dysregulation in carbohydrate metabolism (increased glycolysis/gluconeogenesis and decrease in fatty acid metabolism) are characteristic features for GDM [104]. Overall, the data collected so far suggest that SCFAs may improve insulin sensitivity and reduce the course of inflammatory reactions in several metabolic carbohydrate disorders. For this reason, short-chain fatty acids may be a breakthrough in their use in GDM therapy as novel therapeutics for gestational diabetes.

## 6. The Effect of SCFA on Lipid Metabolism

Lipid metabolism is regulated, inter alia, by SCFAs, thanks to the supply of substrates for lipid synthesis [1]. Acetic and butyric acid are the main substrates for de novo lipogenesis due to their conversion to acetyl-CoA [119]. Coenzyme A is a universal carrier of acyl groups, participating in many enzymatic reactions at the crossing of anabolic and catabolic pathways of fats, sugars, amino acids and nucleotides. Thus, there are indications that SCFAs activate AMP-activated protein kinase (AMPK) [120]. AMPK activation induces signaling pathways and increases the expression of peroxisome proliferator-activated receptor-gamma coactivator-1 alpha (PGC-1-α) in adipose tissue and muscles responsible for regulating the transcriptional activity of many factors, including peroxi-some proliferator-activated receptor alpha (PPAR-α). Activation of PPAR-α lowers the lev-el of triglycerides and is involved in the regulation of energy homeostasis, while PGC-1-α plays a key role in the regulation of cellular energy metabolism [120]. The increase in PPAR-α expression is associated with a reduction in the triglyceride and FFA content. The systemic glucose and lipid homeostasis are also regulated by PPARγ. Depending on the product type, SCFAs influence the AMP/ATP ratio and the activation of AMPK in the liver and muscles, which has a regulatory effect on glucose and lipid metabolism. It has been proved that AMPK positively regulates the lipolysis process by influencing the hormone sensitive lipase (HSL) and adipose triglieride lipase (ATGL) [1]. On the other hand, the antilipolytic effect by inhibiting the phosphorylation activity causing inactivation of HSL in adipose tissue is evident in the conditions of reduced activity of protein kinase A (PKA) [121]. It has been shown that the antilipolytic activity of acetic acid is mediated, inter alia, by its action on HSL [1]. Propionate, in turn, induces intestinal lipolysis by reducing the intracellular content of triglycerides and enhancing the expression of lipolysis genes such as ATGL, HSL and lysosomal acid lipase due to the activation of AMPK phosphorylation and lysine-specific demethylase 1 [122]. HSL and ATGL have been found to be localized in syncytiotrophoblasts, endothelial cells and stromal cells as well [123]. AMPK regulates energy homeostasis at the cellular and whole organism level under stressful conditions. Dysfunction of AMPK correlates with many diseases such as cardiovascular disease, diabetes, inflammatory diseases, and cancer. In other studies, no significant differences in the concentrations of SCFA in the feces and serum in relation to cholesterol concentrations were found, while the concentrations of acetic acid in the feces correlated positively and propionic acid negatively with the levels of intermediate-density lipoprotein cholesterol (IDL-C) [124]. It has been shown that circulating acetic acid is associated with de novo lipogenesis and stimulation of hepatic cholesterogenesis, while propionic acid has an inhibitory effect [5,125].

## 7. The Effect of SCFAs on Lipid Metabolism and Obesity in Pregnancy

The relationship of lipid metabolism with dysbiosis in pregnancy was demonstrated by the studies of Santacruz et al. [126] who observed lower concentrations of Bifidobacterium and Bacteroides strains and higher concentrations of Staphylococcus, Enterobacteriaceae and *Escherichia coli* in obese pregnant women compared to the control group. At the same time, the increase in the entire bacterial population and the Staphylococcus number was associated with an increase in plasma cholesterol concentrations. In turn, increased concentrations of Bacteroides were associated with an increase in HDL-C and a decrease in triglycerides [126]. The above relationships indicate the participation of SCFAs in shaping the lipid metabolism in pregnancy. Although long-chain polyunsaturated fatty acids (LC-PUFA) play a key metabolic role during pregnancy [90], SCFAs appear to be equally important for their role in inflammation as well as for obesity associated with pro-inflammatory adipose tissue status. Lipid metabolism and inflammation are found to be critical processes during pregnancy. It has been shown that maternal diet-induced obesity is associated with the reduction of genes encoding the tightly linked proteins claudin-1 and zonula occludens-1 (ZO-1) in the maternal intestine, which is the most important element in regulating intestinal permeability [87]. This observation suggests that in diet-dependent obesity, SCFAs and their receptors, mainly GPR43, lose their protective effect on maternal gut barrier function. The metabolic effect of GPR43 is mediated through the regulation of energy accumulation and obesity. The presence of these receptors has been demonstrated in white adipose tissue (WAT), including subcutaneous tissue, perirenal, epididymal tissues and adipocytes [31]. In mice, a high fat diet-induced obesity (HFD) induces GPR43 overexpression in WAT, which is not observed in non-obese subjects [88]. SCFAs not only stimulate the adipogenesis process via GPR43 receptors but also inhibit (acetate) lipolysis in a concentration dependent manner [127]. In murine models, the lack of GPR43 induced obesity, while in individuals with overexpression of these receptors only in WAT, body weight was normalized [28]. GPR43-insulin pathway is therefore an important element of the regulation of the body’s energy expenditure, where GPR43 may be a potential tool in the treatment of obesity and diabetes during pregnancy.

## 8. Conclusions

During pregnancy, short-chain fatty acids as products of bacterial metabolism are responsible for maintaining homeostasis in the woman’s body, as well as influencing immune function and carbohydrate and lipid metabolism. SCFAs such as acetate, propionate or butyrate are of particular importance. The activity of SCFAs in the body’s protective processes is manifested by inhibition of histone deacetylase activity and signal transduction through a set of free fatty acid receptors (GPCRs). Metabolic changes occurring during pregnancy contribute significantly to changes in the phylogenetic diversity of the gut microbiota, which differs in several stages of pregnancy.

Dysbiosis of the gut microbiome as a factor associated with metabolic changes, can result in elevated glucose levels and potentially gestational diabetes. Increasing insulin resistance during pregnancy, leading to impaired peripheral insulin signaling, may also be modulated by SCFAs. Women with gestational diabetes have different gut microbiota in late pregnancy compared to those without GDM. Knowledge of possible gut microbiota-host interactions in GDM may provide a potential therapeutic target for improving health outcomes in women with GDM and gut microbiota can serve as an early biomarker for GDM. In situations where lifestyle modification, diet, and exercise are ineffective, supplementation with multi-strain probiotics comprising Bifidobacterium and Lactobacillus can modulate the composition of the gut microbiota and maintain normal ratios of SCFAs concentrations, thus improving health outcomes.

The gut microbiota via SCFAs may regulate blood pressure both early and later in pregnancy. In overweight and obese pregnant women, butyrate produced by the intestinal microbiota may be of particular importance as its concentrations have been observed to negatively correlate with blood pressure and plasminogen activator inhibitor-1 levels. Diet-related overweight and obesity in pregnancy affect the structure of the intestinal microbiota in newborns via vertical transfer of the microbiota and/or its metabolites. The transplacental influence of maternal bacteria on the developing fetus occurs through various mechanisms, including transplacental transport of gut bacterial components, cytokine signaling, and immune programming through metabolites produced by gut bacteria. However, the role of gut bacteria and their metabolites in fetal development is still not fully understood.

The limitation of many studies conducted so far is the use of an animal model (rats, mice), which makes it difficult to relate their results to humans. Despite the evident association of SCFAs with maternal microbiota and metabolism during pregnancy, the above-mentioned relationships need to be confirmed in studies on a large group of humans with considering multifactorial influences.

## 9. Method: Analysis of the Available Literature

**Figure 2:**
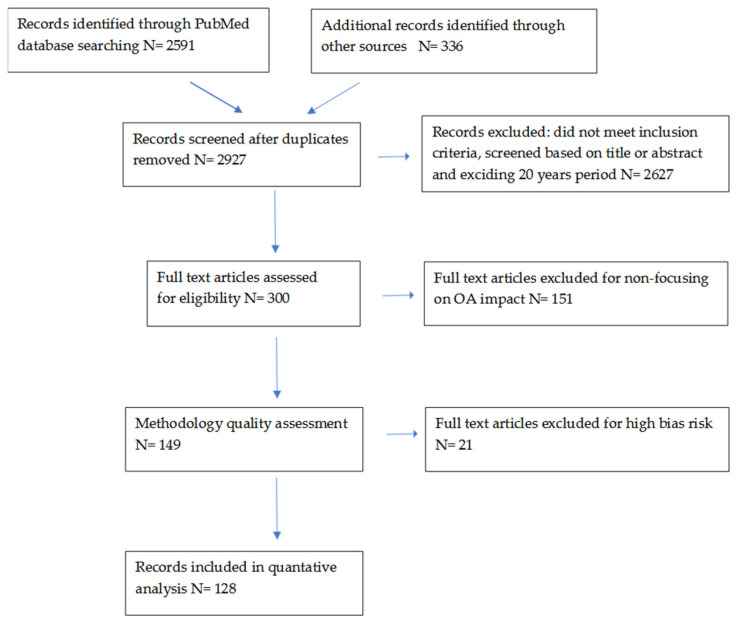
Flow diagram of literature search and selection process.

Search strategy: This study is based on an analysis of available studies and articles (*n* = 2927) that focused on pregnancy and the metabolism of lipids and short chain fatty ac-ids. To this end, the international PubMed records database was searched, and other available sources found in the last 20 years. All articles (*n* = 128) collected through the e-search process used in this article were reviewed by at least two authors. Articles unrelated to the main topic, items duplicated in the databases (PubMed and others), conference summaries, articles written in a non-English language (*n* = 2778) were excluded from the analysis. In our review, only the current full-text studies looking at the relationship be-tween pregnancy, lipid metabolism and SCFA were considered. The study was conducted using a combination of the following keywords: “pregnancy”, “lipids”, “SCFA” in combination with “diabetes”, “hypertension”, “microbiota”. In the case of duplicating information in publications, the ones that most contribute to the analyzed topic were selected. Flow diagram of literature search and selection process is shown below (Figure 2).

## Data Availability

Not applicable.
